# Liquid Biopsies in Progressing Metastatic Colorectal Cancer- Application and their Therapeutic Implications According to the RAS Status

**DOI:** 10.7759/cureus.7035

**Published:** 2020-02-18

**Authors:** José Pereira, Fatima Alves, Filipa Ferreira, Leonor Vasconcelos de Matos, Ana Massena, Ana Martins

**Affiliations:** 1 Medical Oncology, Centro Hospitalar Lisboa Ocidental, Lisbon, PRT; 2 Medical Oncology, Centro Hospitalar Lisboa Ocidental, Lisboa, PRT

**Keywords:** cancer, colorectal, metastatic, liquid biopsy, egfr, ras, oncology

## Abstract

Introduction

The treatment of metastatic colorectal cancer (mCRC) now includes therapy with biological agents inthe first line of treatment. The advances of our knowledge in molecular biology of these tumors allowed the identification of signaling pathways involved in tumorigenesis as potential therapeutic targets. In this field, monoclonal antibodies against epidermal growth factor receptor (anti-EGFR) added to a chemotherapy doublet have demonstrated improved overall survival for these patients. However, mutations in oncogenes NRAS/KRAS are predictive of absence of response to these treatments. Therefore, genotyping in mCRC is essential to personalized treatment. It is known that tumoral heterogeneity and selective pression by targeted therapies can lead to changes in RAS mutational status, along the course of the disease. This opens the possibility of different targeted therapies. Tumor analysis through liquid biopsies allows for the detection of genetic alterations in a less invasive way than common solid tumor biopsy and is currently being validated in different settings, with promising results in mCRC. The main goal of this study was to assess therapeutic implications of Liquid Biopsy (LB) in treatment of progressive mCRC and its potential impact on survival.

Material and methods

A retrospective, observational, unicentric study of patients diagnosed with progressive mCRC and who underwent LB after several lines of treatment, was performed. Analysis of patient and tumor characteristics, as well as LB results was performed with descriptive statistics and survival analysis according to Kaplan-Meier methods and COX analysis with STATA/IC software.

Results

We included 18 patients on whom LB were performed (median age 61 years; 55% (n=10) men). The median follow-up was 37.4 months. At diagnosis, 12 patients had a KRAS mutation. In the LB reassessment, there was a change in the RAS status in six patients, who initially had a mutation and later showed KRASwt (wild type RAS). LB led to a change in the therapeutic plan in these six patients, allowing the use of anti-EGFR therapy. Progression Free Survival (PFS) and Overall Survival (OS) could not be calculated at this time.

Conclusion

LB can revolutionize the approach to mCRC by optimizing therapeutic sequencing in a continuum of care strategy. The search for genetic changes over the course of the disease allows a better therapeutic approach to each patient. In the study presented, the realization of LB allowed an increase in therapeutic options in 1/3 of the patients. It is important to continue these studies with larger samples in order to better validate this strategy.

## Introduction

Cancer is a disease characterized by uncontrolled division and survival of atypical cells. When this growth occurs in the colon or rectum, it is called colorectal cancer (CRC) [1]. CRC is the second most common cancer worldwide and represents 13.2% of all cancers in men and 12.7% in women [1]. According to Globocan 2018, 5645 new cases of colon cancer and 4447 new cases of rectal cancer were diagnosed in Portugal [2]. Metastatic disease at diagnosis occurs in about 25% of cases. However, despite the fact that the majority of patients diagnosed with CRC present localized disease at diagnosis, about 50% will develop metastases in the course of the disease [1].

CRC therapy is variable and depends on the stage of the disease. Surgery is part of the treatment of patients in a less advanced disease, and may be followed by adjuvant or preceded by neoadjuvant chemotherapy. For patients with metastatic CRC (mCRC), therapeutic options are more limited and essentially encompass systemic therapies. The repertoire of treatments available at this stage of disease now includes therapy with biological agents. These include monoclonal antibodies to the Epidermal Growth Factor Receptor (EGFR) such as Cetuximab and Panitumumab, and antiangiogenic agents such as Bevacizumab and Ramucirumab, as well as Tyrosine Kinase inhibitors (TKI) such as Regorafenib [3].

It is thought that there are specific genetic changes that lead to the transformation of the colorectal epithelium into invasive carcinoma. These changes may be inherited or acquired. In addition to the molecular pathways already identified, as implicated in the genesis of CRC, specific molecular changes are also known in oncogenes, tumor suppressor genes and mismatch repair (MMR) genes, as well as hypo- and hypermethylation epigenetic phenomena that are involved in the pathogenesis of CRC [4].

Among the oncogenes involved in sporadic CRC, the most relevant is the RAS oncogene. It exists in 3 cell variants: HRAS, NRAS and KRAS, the KRAS and NRAS being the most frequently mutated in CRC at the level of codons 12, 13, or 61. The RAS genes encode a family of proteins involved in nuclear transduction by activation via MAPK independently of EGFR, leading to the activation of cell proliferation [3]. The identification of RAS mutations has clinical relevance and diagnostic/therapeutic implications since the presence of an NRAS/KRAS mutation in CRC is associated and is predictive of the lack of response to anti-EGFR therapy [1]. The V600E variant of the BRAF gene appears to be a negative prognostic marker, but is present in a minority of tumors and is mutually exclusive with the presence of a RAS mutation. Although several other biomarkers are being studied as potentially predictive of response to therapy in mCRC, currently only RAS mutations and tumor location (left vs. right) are robust factors to be taken into account when selecting patients for anti-EGFR [1].

EGFR is a tyrosine kinase receptor, which is activated by binding its extracellular ligand. This link activates the intracellular signaling pathways via the RAS/ RAF/MAPK, STAT and PI3K/AKT pathways, modulating cell proliferation, adhesion, angiogenesis, migration and survival. [3] This receptor is a therapeutic target in mCRC. However, due to its predictive value of the lack of response to anti-EGFR therapy, the RAS status allows for the selection of patients who may benefit from therapy with these agents. The CRYSTAL trial demonstrated the benefit of adding Cetuximab to chemotherapy in patients with wild-type RAS (RASwt) mCRC, leading to increased in Progression Free Survival (PFS), rate of global response and Overall Survival (OS) [5]. In the PRIME trial, the addition of Panitumumab to chemotherapy significantly improved the OS when compared to chemotherapy alone in KRASwt mCRC patients. Encouragingly, the average overall survival (OS) of the most recent phase III trials in the mCRC RASwt population currently exceeds 30 months [6-9]. However, these patients invariably show progression of their disease. This is partly due to the emergence of mutations that occur in genes in the RAS pathway during treatment, and to the selection of subclones resistant to therapy [10]. The shift from a RASwt status to a mutated RAS, which can occur in mCRC during treatment with anti-EGFR, can lead to the development of resistance to anti-EGFR by decreasing the population of RASwt clones while pre-existing subclones with RAS mutation proliferate. The opposite event, of selection of wt clones in patients with RAS mutation, has also been studied and shown to occur with a prevalence of about 30% [11]. Other mutations, such as in the BRAF gene, may be subject to the same principles of tumor heterogeneity and dynamic selection by therapeutic pressure. Another mechanism of resistance which occurs in about 25% of patients treated with anti-EGFR therapies is the mutation of the EGFR extracellular domain, which prevents binding of monoclonal antibodies. 

A continuum of care perspective, in the treatment of patients with mCRC requires the optimization of available therapies, making the most of therapeutic lines available and incorporating multimodal strategies. Considering the predominance of specific tumor subclones dictated by the selective pressure of the target therapies, the knowledge of the mutational changes that occur during the treatment may optimize the therapeutic options for these patients. Depending on the location of the metastatic lesions, traditional tissue biopsy can be too invasive and risky. Currently, with liquid biopsies (LB), it has become possible to search for genetic changes in a less invasive way for the patient.

Liquid biopsies are a blood test that allows the detection of circulating tumor cells (CTC) and/or small fragments of circulating tumor DNA (ctDNA) [10]. This test allows the non-invasive characterization of the tumor cell genome [12]. This new technology may revolutionize the treatment of cancer patients as it allows for the non-invasive determination of tumor heterogeneity with important therapeutic implications [10]. In mCRC, >75% of patients have detectable ctDNA. The analysis of these circulating tumor cells showed a correlation with the prognosis of the disease, also showing a great agreement with tissue biopsies [13-15].

The main objective of this study is to understand the therapeutic impact of the performance of LB in patients with progressing mCRC and the need of a new therapeutic line.

## Materials and methods

This observational, single-center retrospective study included patients with a diagnosis of mCRC and after 2 or more lines of treatment, were selected to perform liquid biopsies. All patients with mCRC who were selected to undergo LB, from June 2018 to June 2019, were evaluated. One LB was performed by patient. The inclusion criteria required were: age> 18 years, CRC confirmed by histology, metastatic disease with confirmed progression according to RECIST 1.1 criteria, existence of analysis of the RAS status in a solid sample (tumor or metastasis) prior to the performance of LB and previous use of therapy with anti-vascular edothelial growth factor (VEGF) or anti-EGFR agents, and Eastern Cooperative Oncology Group / Performance Status (ECOG / PS) 0-1. Patients who refused to undergo LB were excluded.

During this period, 18 patients underwent LB in our center with a proven diagnosis of mCRC. From the information present in the clinical process, the following data were collected: age, sex, initial diagnosis data, initial stage, primary tumor location (left/right colon), initial metastasis data, metastasis site, follow-up time, RAS status, LB date, previous therapeutic regimens, LB result and therapy performed after LB result.

Statistical analysis

The analysis of the patient's demographic characteristics, tumor and previous therapies as well as the results of LB was performed using descriptive statistics. Survival analysis was performed according to Cox proportional hazards model, with hazard ratio (HR) calculation and 95% confidence intervals. Survival curves were calculated according to Kaplan-Meier methods. Statistical analysis was performed using the software Stata/IC and all results with a p-value of less than 0.05 were considered statistically significant.

## Results

A total of 18 patients were eligible for this study (median age was 61 years; 56% (n = 10) male). The median follow-up was 37.4 months (11-106). The characteristics of the sample are shown in Table 1. The vast majority of patients (69.2%, n = 14) had a tumor at the level of the left colon. Half of the patients had synchronous metastasis and half had metachronous metastasis. Most patients had metastasis at 2 or more sites (n = 12), with the liver being the most frequent site of metastasis (n = 13) followed by the lung (n= 8).

**Table 1:** Sample characterization

	Variables	N=18	%
Gender	Male	10	55.6
	Female	8	44.4
Median age at diagnosis (min, max)		61(30, 78)	
ECOG/PS at diagnosis	0	17	94.4
	1	1	5.6
Primary tumor location	Right colon (RC)	4/13	30.8
	Left colon (LC)	9/13	69.2
	Rectum ( R)	5	27.8
Grade of diferentiation	1	6	33.3
	2	10	55.6
	3	2	11.1
Lymph nodes involved at diagnosis	0	6	33.3
	≥ 1	12	66.6
Metastisation	Synchronous	9	50
	Metachronous	9	50
	Multiple (≥2 locations)	12	66.6

The initial RAS status was investigated in the surgical resection specimen in 12 patients, in primary tumor biopsy in 5 patients and in metastasis biopsy in 1 patient. Initially, 12 patients had a KRAS mutation at diagnosis. The median interval between the initial biopsy at diagnosis and the performance of LB was 39.5 months (9-100). Six patients showed a difference in the level of the RAS mutation in LB, and of these, all of them initially presented KRAS mutation and later were wild type for this mutation (Table 2).

**Table 2:** Tumor location and RAS status (Solid Biopsy vs. Liquid Biopsy) LB - liquid Biopsy; WT - wild type; mut - mutated

Sample of the initial mutational study	Inicial Status RAS	LB RAS Status	Conversion?	Months from diagnosis to LB (months)
Primary tumor biopsy	KRAS	WT	Yes	9
surgical resection specimen	WT	WT	No	64
Primary tumor biopsy	WT	WT	No	25
surgical resection specimen	WT	WT	No	78
surgical resection specimen	KRAS	KRAS	No	50
surgical resection specimen	KRAS	WT	Yes	36
surgical resection specimen	KRAS	WT	Yes	57
surgical resection specimen	WT	WT	No	100
surgical resection specimen	WT	WT	No	17
surgical resection specimen	WT	WT	No	88
surgical resection specimen	KRAS	WT	Yes	67
Primary tumor biopsy	KRAS	KRAS mut	No	14
surgical resection specimen	KRAS	KRAS mut	No	15
Primary tumor biopsy	KRAS	WT	Yes	26
surgical resection specimen	KRAS	KRAS mut	No	33
Primary tumor biopsy	KRAS	KRAS mut	No	17
surgical resection specimen	KRAS	KRAS mut	No	43
Biopsy of metastasis	KRAS	WT	Yes	94

Three groups were defined according to the LB result, depending on the maintenance of the initial status or conversion (Table 3)

**Table 3:** Dividing the sample into subgroups, depending on the variation of the RAS status

Subgroup 1	Subgroup 2	Subgroup 3
RASmut status Maintenance	RASwt status Maintenance	RASmut to wt conversion
6 patients	6 patients	6 patients

Subgroup 1 - Maintenance of RASmut status

This subgroup is quite homogeneous in terms of tumor location as well as metastasis pattern. Of these, two patients had neoplasms of the right colon, two of the left colon and two of the rectum. Three patients had synchronous metastasis and three had metachronous metastasis. Four patients underwent anti-VEGF therapy, and after LB, three patients were offered anti-VEGF therapy again. Of the patients in this group, four deaths were verified, with an overall survival of 31 months (table 4).

**Table 4:** Sample characterization - SubGroup1 LB - liquid biopsy

Variables	N=6
Location of primary tumor	
Right colon	2
Left colon	2
Rectum	2
Mestastization	
Sínchronous	3
Metáchronous	3
multiple (< 2 locations)	0
Metastization location	
Hepatic	5
Pulmonar	1
Ganglionar	4
Peritoneal	1
Other (ovary)	1
Treatments before LB	
Paliative chemotherapy	
1 line	2
2 lines	3
4 lines	1
Anti-VEGF therapy	4

Subgroup 2 - Maintenance of RASwt status

This group was also homogeneous. No patients with RC were in this group. Regarding the location of the primary tumor, three patients had a tumor at the level of the left colon and three patients at the rectum. In terms of metastasis pattern, the results were also homogeneous with three patients presenting synchronous metastasis and three patients with metachronous metastasis. In terms of chemotherapy, three patients underwent adjuvant therapy after surgery, and all underwent at least one line of palliative chemotherapy. For biological therapy, three patients underwent anti-EGFR therapy and three patients under anti-VEGF therapy, of which two patients underwent sequential therapy (anti-EGFR - anti-VEGF), one underwent only anti-VEGF therapy and one only anti-EGFR therapy. In this group, there were no deaths (table 5).

**Table 5:** Sample characterization - SubGroup2

Variables	N=6
Location of primary tumor	
Right colon	0
Left colon	3
Rectum	3
Metastization	
Synchronous	3
Metáchronous	3
multiple (< 2 locations)	3
Metastization location	
Hepatic	3
Pulmonary	3
Bone	1
Other	1
Treatment before LB	
Adjuvant Chemotherapy	3
Paliative chemotherapy	
1 line	2
2 lines	1
3 lines	1
4 lines	2
anti-EGFR therapy	3
anti-VEGF therapy	3

Subgroup 3 - Conversion RASmut to RASwt

In this subgroup of patients, all had the primary neoplasia located at the colon with the majority being on the left colon (n = 4). In terms of metastasis, the sample was homogeneous with the same proportion of patients with synchronous and metachronous metastasis. The initial RAS study was performed in the surgical specimen in three patients, by biopsy of the main tumor in two patients and in one case by metastasis biopsy. It should be noted that the median interval between the first sample for the RAS study and the LB was 46.5 months (9-94), this interval being greater than in the other subgroups.

After the LB result indicating a change in the RAS mutational status, three patients were started chemotherapy with anti-EGFR, given that the current mutational status is wild type. Note that three patients were not offered this therapy due to the following: patient #1 - to reserve anti-EGFR for subsequent lines (having maintained anti-VEGF); patient #2 - considered to have no benefit in initiating therapy due to the deterioration of the clinical condition and patient #4 - had a stable disease at the time, reserving anti-EGFR for subsequent lines (table 6).

**Table 6:** Sample characterization - SubGroup3 RC -Right Colon; LC - Left Colon; mut - mutated, wt - wild type; M - male; F -female

ID	gender	age	location	Initial RAS	metastization	Multiple metastization (more than 2)	Location of metastasis	Chemotherapy More than 3 lines?	RAS LB
# 1	M	61	RC	RASmut	Synchronous	YES	Hepatic + Ganglionic	NO	RAS WT
# 2	F	78	LC	RASmut	Synchronous	YES	Hepatic + Pulmonary	YES	RAS WT
# 3	F	43	LC	RASmut	Metachronous	YES	Hepatic + Pulmonary + Peritoneal	YES	RAS WT
# 4	M	65	RC	RASmut	Metachronous	NO	Hepatic	YES	RAS WT
# 5	F	69	LC	RASmut	Synchronous	NO	Hepatic	NO	RAS WT
# 6	M	51	LC	RASmut	Metachronous	YES	Pulmonary + Peritoneal	NO	RAS WT

Survival analysis

Due to the reduced sample and limited follow-up time, the median survival has not yet been reached, when population survival analysis is performed, according to the mutational status conversion. Thus, data on overall survival or progression-free survival are not obtainable in this initial analysis.

## Discussion

Liquid biopsies can change the way patients are managed. The monitoring of potential genetic changes through LB can allow the identification of relevant biomarkers in the assessment of disease recurrence, resistance to therapy and response to treatment. It may also lead to a better adaptation of the therapy to the most current characteristics of the tumor [12].

In the analysis, it was found that in 1/3 (n = 6) of the patients there was a change in the initial RAS status (from RASmut to RASwt status) during the course of the disease. This mutational change has an important impact on the therapeutic options available to these patients since it allows the use of anti-EGFR monoclonal antibodies, which is ineffective in patients with the RASmut genotype.

In this subgroup of patients who initially had RASmut, all underwent anti-VEGF therapy treatment. Preclinical observations suggest that hypoxia may negatively select RASmut clones, favoring the prevalence of RASwt clones [14]. This fact may explain the “conversion” observed in this subgroup.

Bearing in mind the high known correlation between tissue sample evaluation (liver metastases) with LB, it is worth noting that two patients with RAS status conversion did not present disease secondary to this organ, which may contribute to potential false negatives [15].

The performance of LB allowed to expand the therapeutic weapons available to treat six patients (1/3) of the sample. As they presented with a RASwt mutational status, the possibility of administering anti-EGFR therapy was opened. Of the six patients in this subgroup, three started systemic chemotherapy together with anti-EGFR. In the remaining patients, the possibility of using this class of drugs was admitted after disease progression.

The study presented here, presents a very small sample of patients. This fact is due to the relatively recent introduction of LB in clinical practice. Not being able to draw significant conclusions, namely with regard to survival data, this study provides evidence of the clinical applicability in the real world of LB which, in the evolutionary course of mCRC, may open therapeutic windows with valid clinical repercussions for patients.

## Conclusions

LB have been the subject of considerable scientific interest in recent years. Its use can provide a more harmless way to access information about tumor genetic characteristics. Being less invasive and cost effective, it can be used more frequently than biopsies in solid pieces, leading to its possible implementation in interim evaluations and after disease progression. It is known that the amount of circulating DNA may be related to the tumor burden and be a possible marker of response to instituted therapies. The fact that it allows the analysis of a potential continuum of genetic alterations throughout the course of the disease may contribute to the optimization of personalized and individualized therapeutic sequencing of patients with mCRC.
